# Craniosacral Therapy Use in Normal Pressure Hydrocephalus

**DOI:** 10.7759/cureus.14886

**Published:** 2021-05-07

**Authors:** Young Park, Jacob Kabariti, Leonid Tafler

**Affiliations:** 1 Family Medicine, Touro College of Osteopathic Medicine, New York, USA; 2 Primary Care, Touro College of Osteopathic Medicine, New York, USA

**Keywords:** normal pressure hydrocephalus, hydrocephalus, csf shunt, csf drainage, lumbar puncture, osteopathic manipulative medicine, omm, craniosacral therapy, cv4, venous sinus drainage

## Abstract

Nearly 700,000 adults in the US have normal pressure hydrocephalus (NPH), but it is often misdiagnosed as Alzheimer’s or Parkinson’s disease. In fact, a small percentage of people with the disease are properly diagnosed. NPH presents classically with a triad of symptoms: ataxic gait, dementia, and urinary incontinence. Diagnosis and treatment are provided together through a lumbar puncture. However, the only effective treatment that exists is a shunt insertion, which is a highly invasive procedure with uncertain responsiveness. As NPH is primarily diagnosed in those in advanced ages (60s and 70s), adjunctive treatment modalities should be further studied. Here we present a case of a patient diagnosed by a neurosurgeon and neurologist with NPH and a candidate for a shunt insertion whose symptoms substantially improved with one month of osteopathic manipulative treatment. Osteopathic considerations and literature are also reviewed in the broader context of craniosacral treatment.

## Introduction

Normal pressure hydrocephalus (NPH) is highly prevalent, but only 20% cases are diagnosed properly [1]. It is caused by impaired cerebrospinal fluid (CSF) absorption that results in CSF accumulation. It is often idiopathic and most commonly in adults older than 60 years of age. NPH can also occur due to obstruction and fibrosis of subarachnoid villi secondary to inflammatory diseases of the central nervous system, intraventricular hemorrhage, or subarachnoidal hemorrhage. It presents classically with a triad of symptoms: ataxic gait, dementia, and urinary incontinence [2]. Ataxic gait is the most prominent clinical feature, but not all features are always present [3].

Diagnosis of NPH includes an MRI that must show ventriculomegaly without sulcal enlargement. To confirm the diagnosis, a lumbar puncture (LP) is needed. The severity of symptoms must be assessed before and after the LP. The LP would indicate normal or only slightly elevated intracranial pressure. Upon removal of 30-50 mL of CSF, an improvement of symptoms provides confirmation of the diagnosis of NPH. Additionally, the removal of CSF also alleviates symptoms [3].

Shunt surgery is only recommended for those patients with a high likelihood of responding to shunt surgery through predictive tests due to extraordinarily high failure rates. Nearly 16% of shunts in adults fail over six years [4]. Thus, only a fraction of patients with NPH receive shunt surgery, and only 60%-80% improve following shunt surgery [5]. According to the published study in the journal Neurosurgery in 2005, only 11,000 were treated for NPH [6]. Furthermore, complications include mechanical malfunctions on the shunt, blockages, and infections [3]. Here we present a case of NPH that was diagnosed and referred for shunt surgery. Due to the patient's hesitancy toward an invasive procedure, the patient came to his primary care osteopathic physician seeking an alternative therapy. He was offered craniosacral osteopathic manipulative treatment, which has been proven to influence the flow of CSF. It was performed, and the patient’s symptoms noticeably improved as confirmed by the patient and his family.

## Case presentation

A 78-year-old male, who had been a patient of the osteopathic family clinic for over 10 years, presented with his wife to discuss a pre-surgical assessment for the placement of an intracranial shunt. He had a past medical history of diabetes mellitus, hypertension, hyperlipidemia, and a stroke in 2013. The stroke resulted in left hemiparesis affecting the lower extremities more than the upper extremities but was followed by a complete recovery. For the past two years, he developed a progressive short-stepped shuffling gait with hesitancy occurring with turning and swaying only to the right side while walking. Additionally, he described nocturia with increased bladder frequency and urgency and subjective memory difficulty without any mental impairment. The patient ambulated with a cane and stated that his biggest concern was swaying to the right with a fear of falling. He described difficulty in turning and freezing due to this feeling of loss of balance. The patient denied dyskinesias, hallucinations, nightmares, tremors, headache, or nausea. His most recent MRI suggested stable mild-to-moderate diffuse ventriculomegaly. He was referred to a neurologist at NYU Langone who diagnosed NPH and subsequently was referred to a neurosurgeon at Columbia Presbyterian who deemed the patient a candidate for a surgical shunt.

Due to his hesitancy toward the recommended invasive procedure, the patient agreed to try craniosacral therapy to relieve the NPH prior to getting the shunt recommended by the neurosurgeon. The patient tolerated the first osteopathic therapy session well and returned to the office one week later. He stated that he noticed a significant improvement with his gait two to three days after the first session, but symptoms had slightly returned towards the end of the week. A second craniosacral session was performed, and the patient felt an improvement in his NPH symptoms after the therapy.

Craniosacral therapy sessions were conducted one week apart lasting 15 minutes. Upon the third session, the patient stated that his gait had significantly improved and turned 90 degrees quicker without significant hesitation. The patient continued to ambulate with a cane but was able to walk faster and felt more comfortable in crowded environments which he had not felt before. The patient also stated that his bladder urgency improved slightly over the previous three weeks. The remaining sessions were spaced two weeks apart until only monthly sessions were necessary to help alleviate symptoms concerning gait.

**Figure 1:**
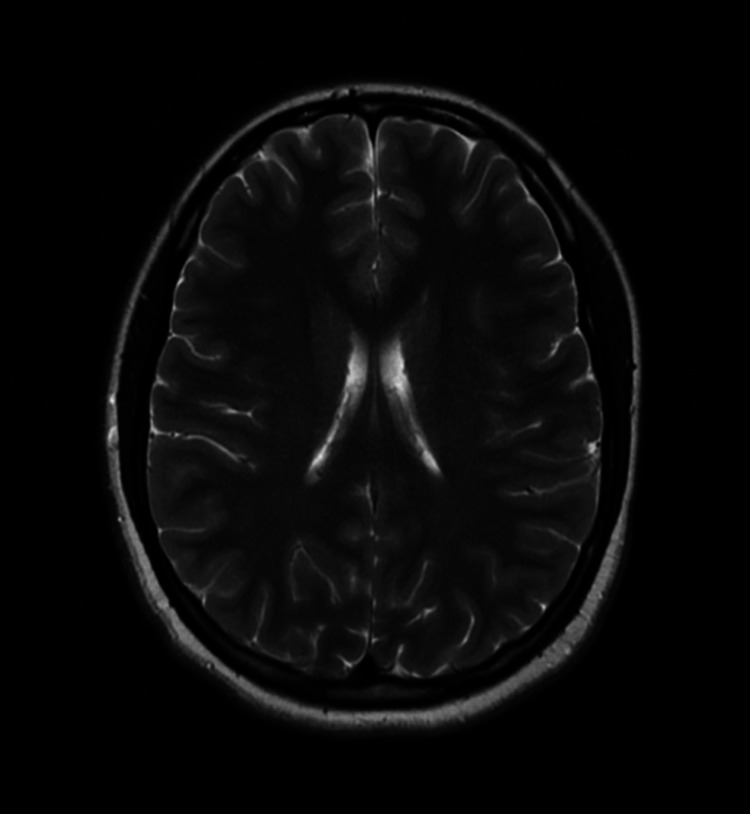
Normal MRI (axial T2) Case courtesy of Associate Professor Frank Gaillard, Radiopaedia.org, rID: 37605

**Figure 2:**
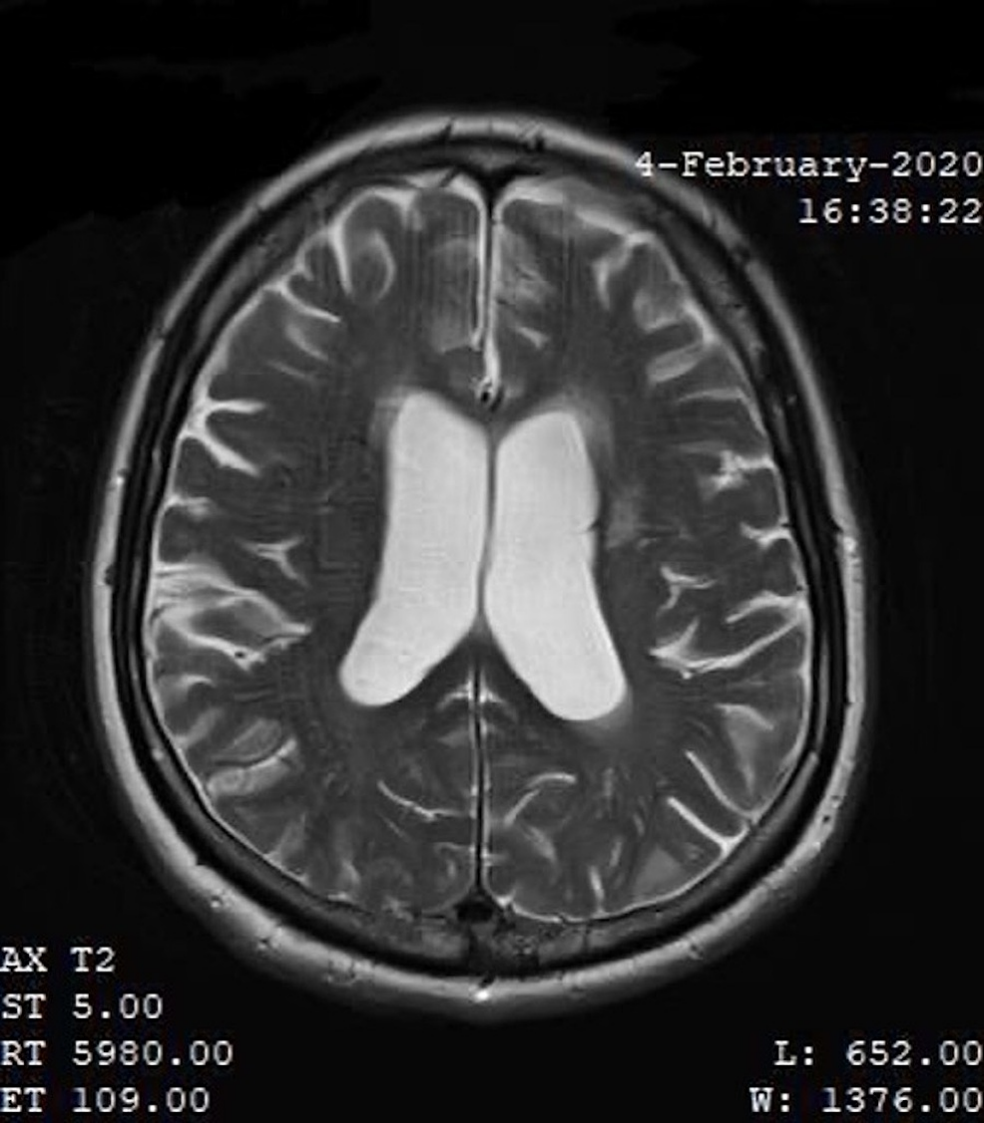
Patient MRI performed on February 4, 2020

**Figure 3:**
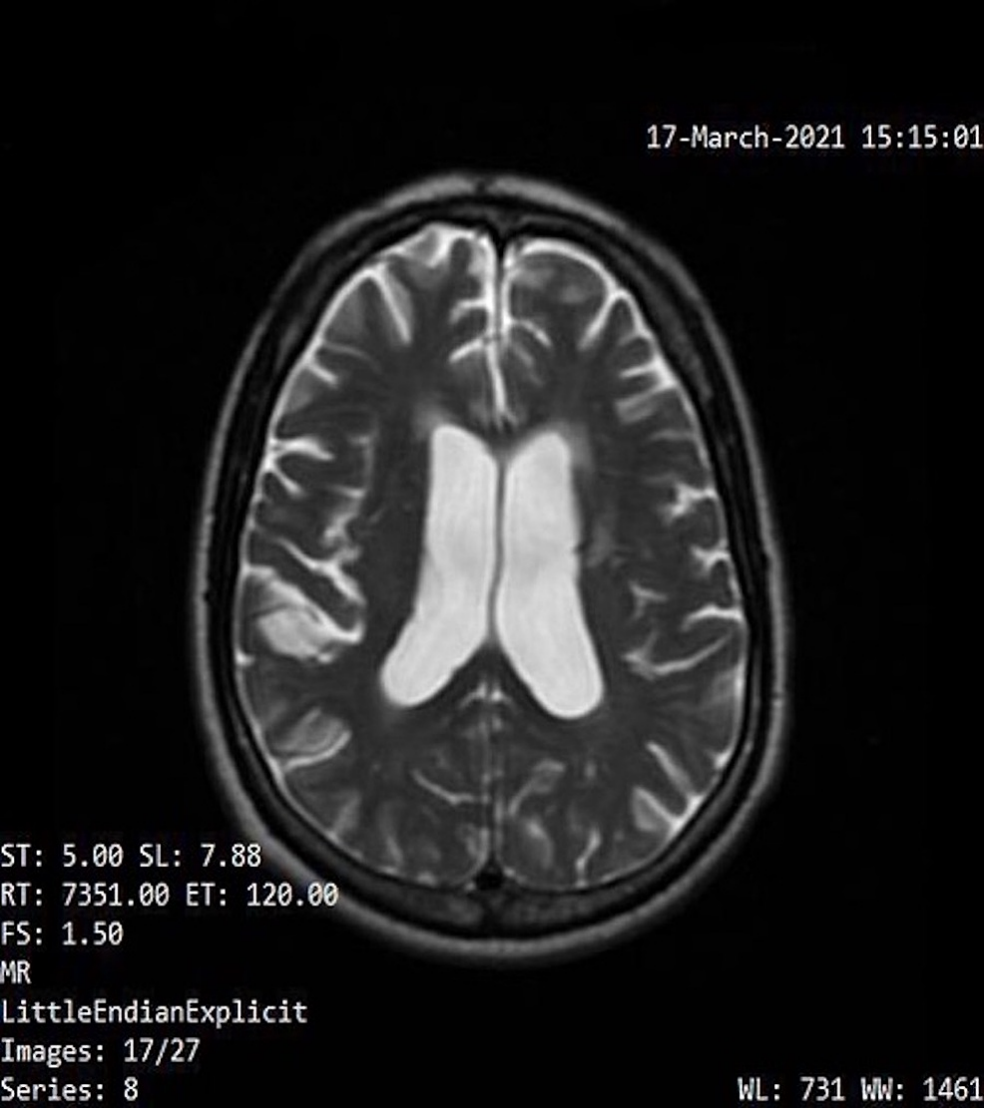
Patient MRI performed on March 17, 2021

An MRI was performed in March 2021, which was three months after the initial relief of symptoms from craniosacral therapy. This was compared to a previous MRI performed in February 2020, which was nine months prior to receiving osteopathic treatment. A neuroradiologist was consulted, and the imaging results showed no significant change in the ventriculomegaly. Yet, the patient agreed that symptoms were diminished after craniosacral therapy. According to a study of patients that received a shunt, imaging plays an important role in the diagnosis and follow-up, but the ventricular size or degree of atrophy on subsequent MRIs does not correlate with patient response and symptoms [7]. A normal axial T2 MRI, the patient’s MRI performed on February 4, 2020, and the patient’s MRI performed on March 17, 2021, are shown in Figures 1-3).

## Discussion

**Figure 4:**
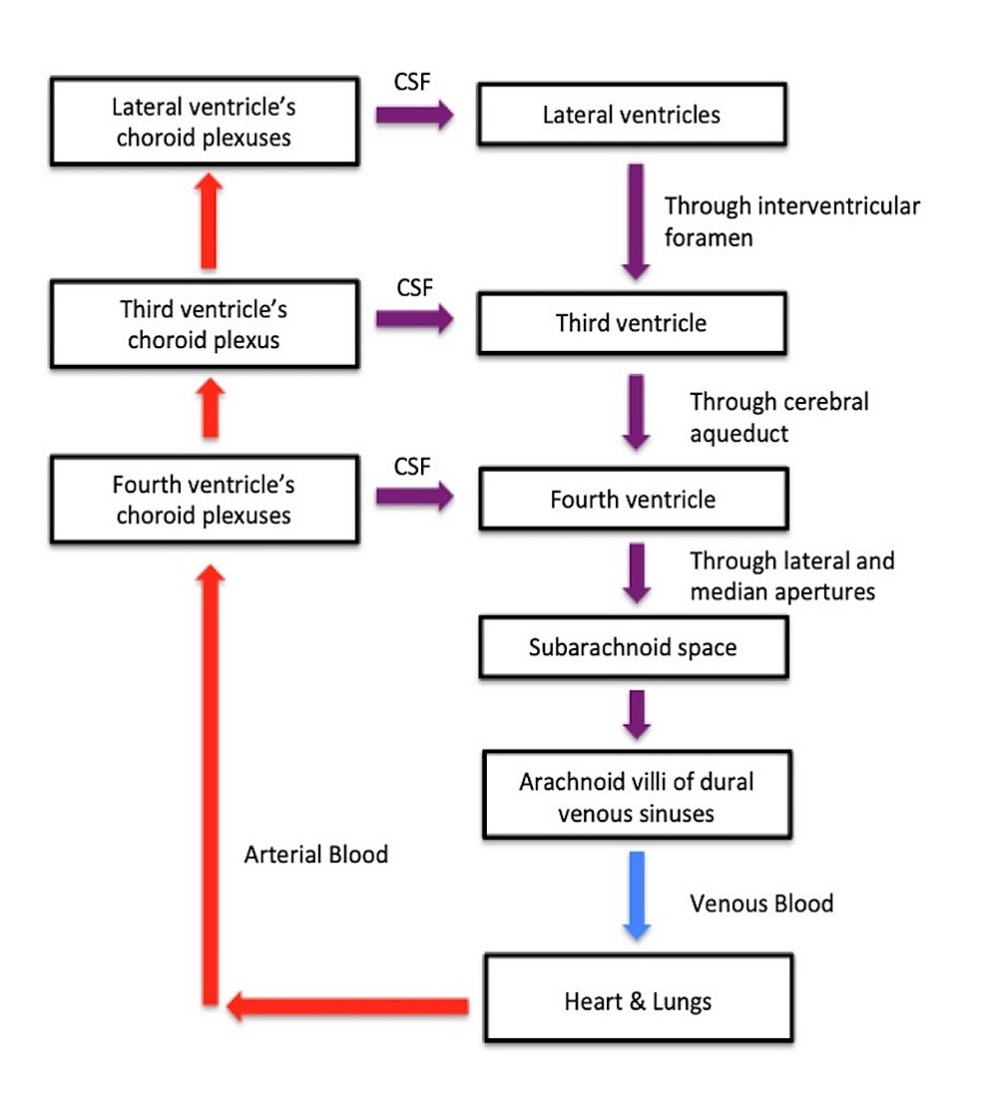
CSF pathway CSF, cerebrospinal fluid

NPH results in an abnormal accumulation of CSF. The CSF is produced in the choroid plexus of the ventricles and moves through a pathway. First, it moves from the lateral ventricles through the interventricular foramina to the third ventricle. The CSF then proceeds through the cerebral aqueduct to the fourth ventricle and through the lateral and median apertures to the subarachnoid space. Finally, it is absorbed into the venous bloodstream via the arachnoid villi of the dural venous sinuses [8]. Any disruption to the natural flow of CSF, such as overproduction or obstruction, may cause a bottleneck in the pathway. The flow of CSF is demonstrated in Figure 4.

Craniosacral therapy is a hands-on and noninvasive technique that utilizes light touch to evaluate and balance restrictions in the craniosacral system that includes, but is not limited to, the bones, nerves, fluids, and connective tissues. Furthermore, the flow of CSF in the fluid-based system resembles a respiratory excursion and can be measured. The optimal rate of the cranial rhythmic impulse is approximately 8-12 cycles per minute. Using a soft touch generally no greater than 5 grams, physicians evaluate the rhythm of CSF for blockages and the positioning of the cranial bones for any restrictions in motion [9]. This can be accomplished utilizing various techniques that make up craniosacral therapy. Contraindications to craniosacral therapy include increased intracranial pressure, intracranial hemorrhage, tumors, and aneurysms.

Compression of the fourth ventricle is a well-known osteopathic technique aimed at influencing the balance between the sympathetic and parasympathetic branches of the nervous system. It has been used successfully to reduce pain, to decrease sleep latency, and as an adjuvant treatment of ADHD [10]. The fourth ventricle lies anterior to the occipital squama, and by applying pressure to the occiput, the physician can influence the cranial rhythmic impulse and help move the CSF thereby relieving the congestion in the ventricles.

**Figure 5:**
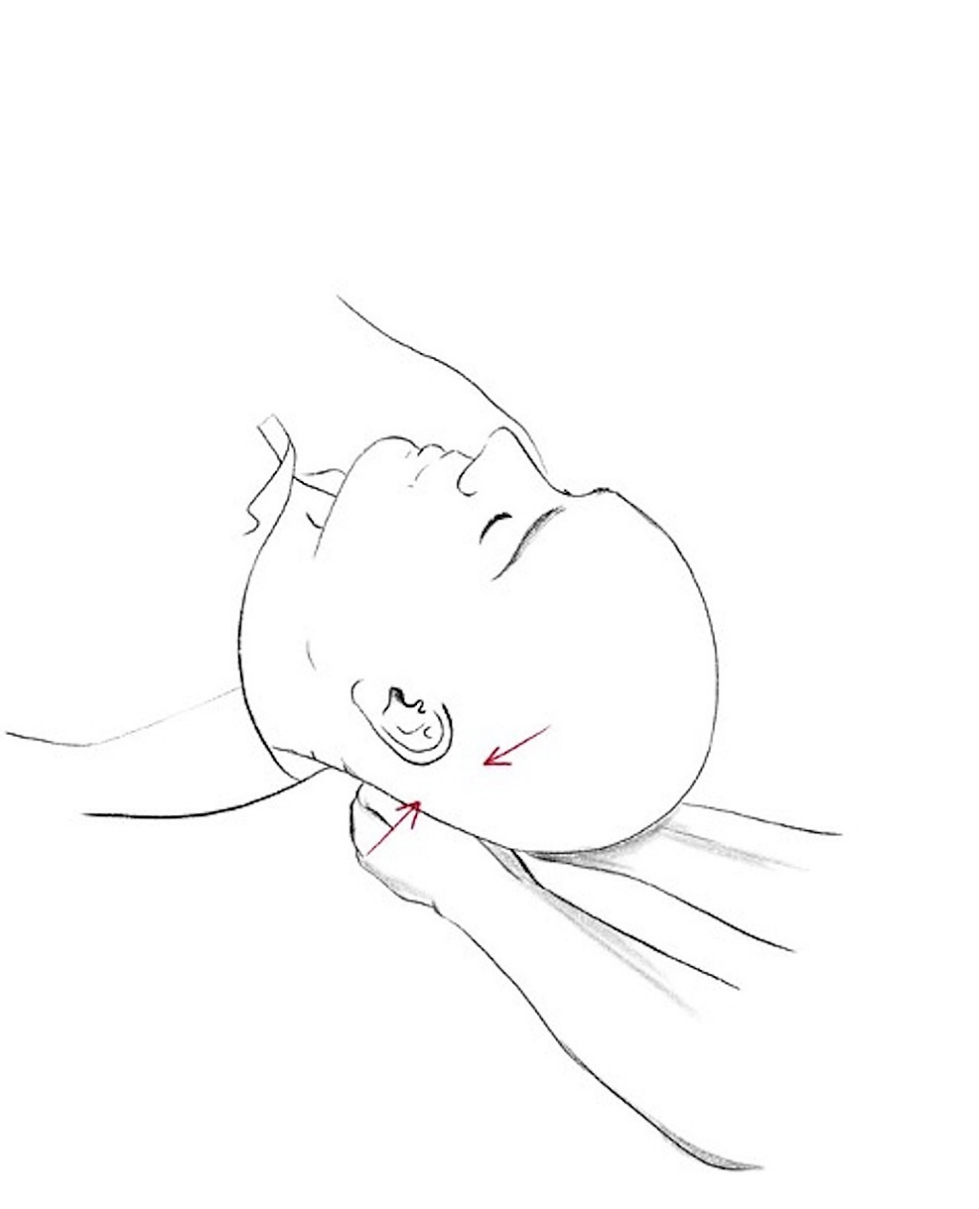
CV4 oblique view

**Figure 6:**
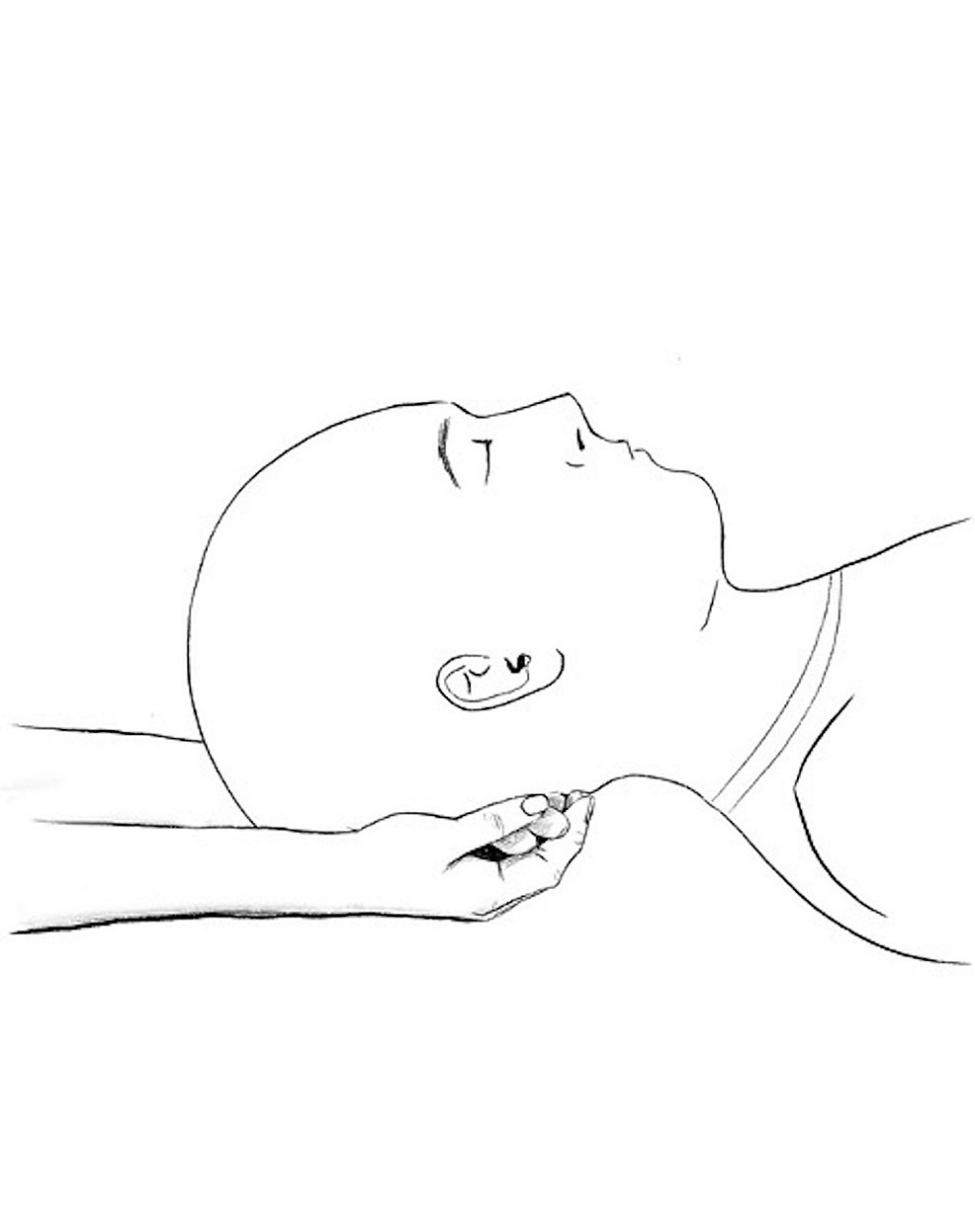
CV4 side view

The compression of the fourth ventricle, also known as the CV4 technique, is done with the patient lying supine and the physician's thenar eminences cradling the occiput. The physician encourages extension towards the physician while discouraging flexion. These movements are continued until a motionless state is reached where a softening and warmth of the surrounding area occurs [9]. This technique is demonstrated in Figures 5, 6.

Venous sinus drainage is also a technique commonly used within craniosacral therapy. The goal of venous sinus drainage is to enhance the flow of venous blood through the venous sinuses that exit the skull through the jugular foramen and return to the central venous circulation. As the CSF is absorbed into the bloodstream through the arachnoid granulations via the superior sagittal sinus, enhancing sinus drainage optimizes CSF reabsorption and prevents accumulation.

**Figure 7:**
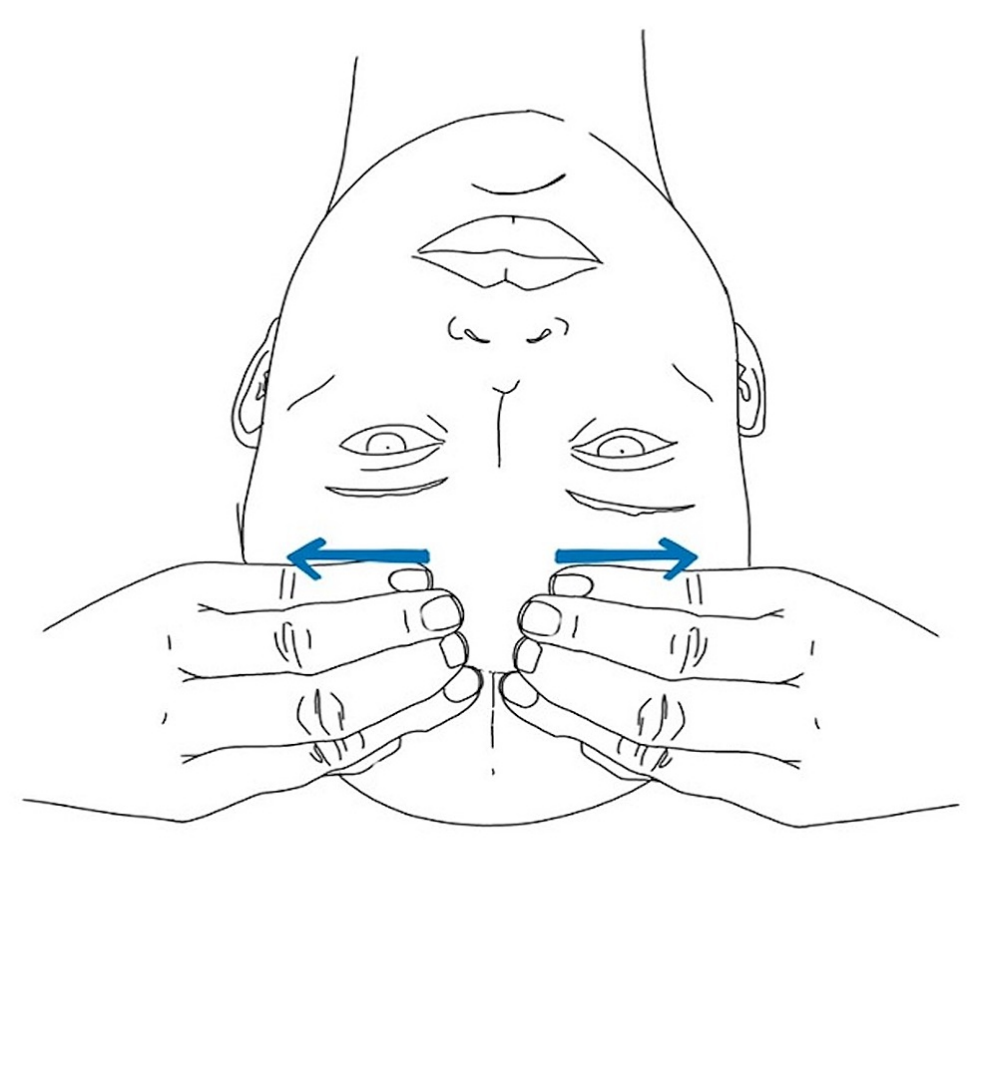
Venous sinus drainage at the distal superior sagittal sinus

**Figure 8:**
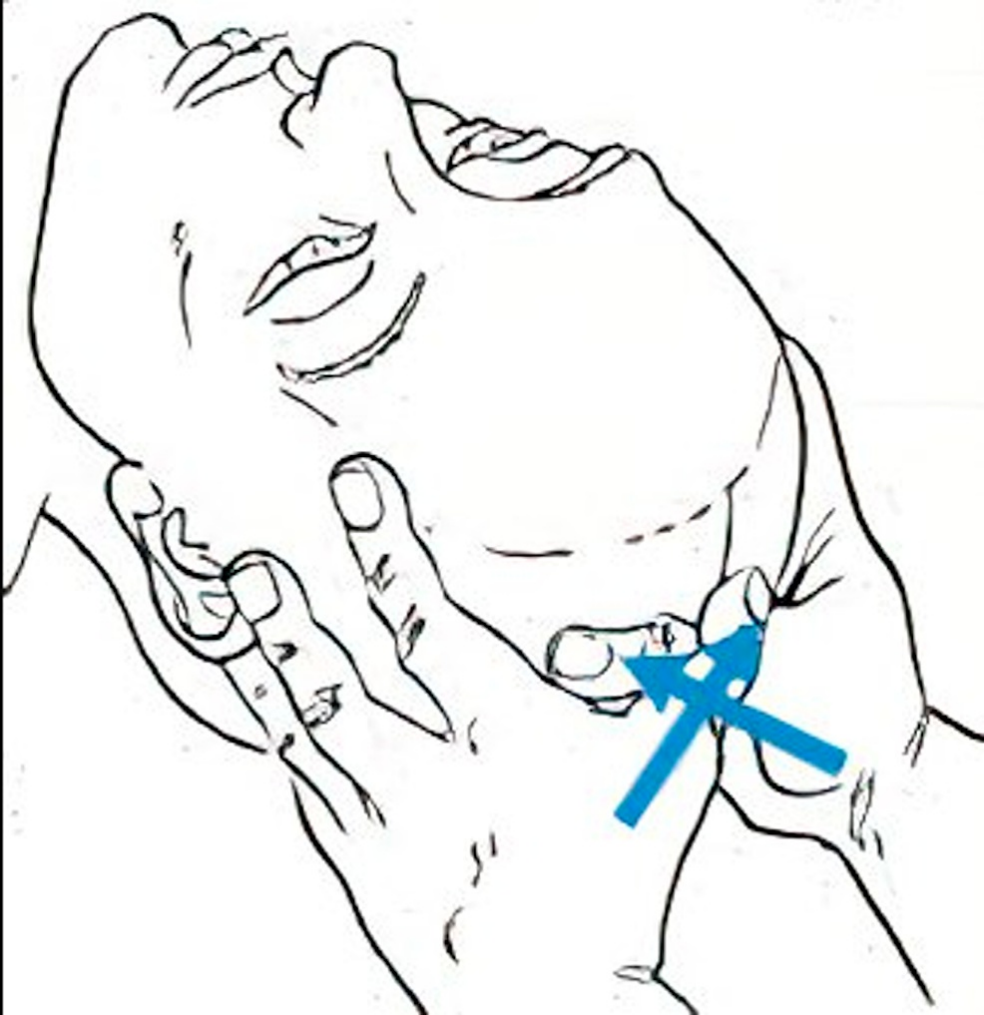
Venous sinus drainage at the proximal superior sagittal sinus

In a study of the efficacy of the venous sinus drainage technique, two groups of 39 participants were recruited. Venous sinus drainage was performed by light touch in one group and a sham therapy with no touch was performed in another group. Results demonstrated improvement in hemodynamics at the cranial base as measured by ultrasonography in the group with true venous sinus drainage compared to the sham therapy without touch [11]. The venous sinus drainage technique is demonstrated in Figures 7, 8.

In the current observational study with a patient diagnosed with NPH, craniosacral therapy was found to alleviate symptoms associated with his diagnosis. Specifically, the CV4 technique influenced the patient’s cranial rhythmic impulse and optimized CSF flow through the ventricles. The CSF is then reabsorbed into the venous blood where the additional venous sinus drainage technique can help. Venous sinus drainage improves the drainage of venous blood flow by manually opening the sinus tracts, helping in the reabsorption of CSF. These two techniques can be combined to improve CSF flow and reabsorption, thereby improving the accumulation of CSF found in NPH.

**Figure 9:**
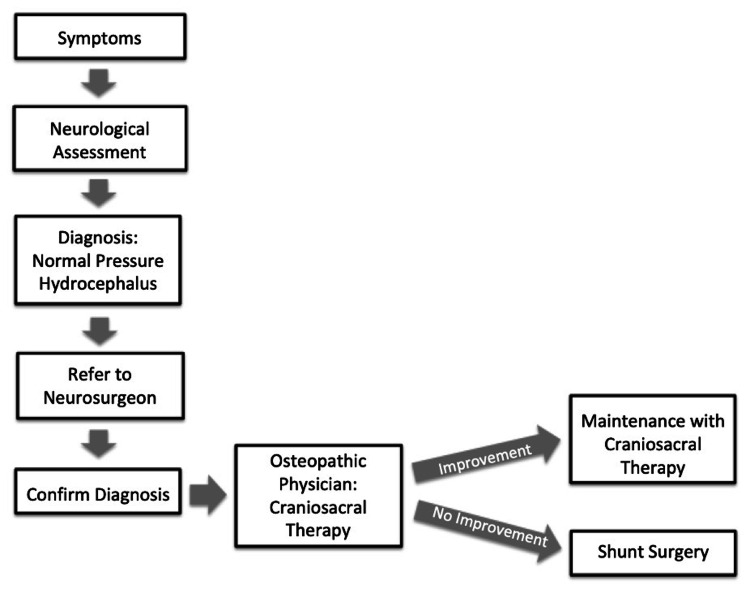
Proposed normal pressure hydrocephalus diagnosis and treatment workflow

Lastly, there is an inherent weakness of a case report representing a single sample. However, due to the overwhelming number of patients seeking alternative treatment to surgery, the importance of a viable treatment in this field is crucial. This report encourages prospective research regarding NPH and the efficacy of non-invasive craniosacral therapy versus observation prior to moving forward with standard shunt therapy. The proposed workflow for the diagnosis and treatment of NPH is shown in Figure 9.

## Conclusions

Due to our patient’s age and hesitancy toward an invasive procedure, he agreed to try craniosacral therapy to relieve NPH prior to getting a shunt as recommended by the neurosurgeon. Initially, the patient was treated with one session. Upon craniosacral therapy to improve the flow of CSF, the patient’s symptoms improved a few days after the first session. Weekly follow-up therapy was performed. After 3.5 weeks, the patient was asymptomatic. Continued monitoring and monthly craniosacral therapy to improve the flow of CSF were performed. In conclusion, craniosacral therapy can be utilized in relieving a patient’s NPH symptoms. Performed at regular intervals, it was shown to be an effective treatment that can be used in a stepwise approach prior to a more invasive treatment such as a CSF shunt.
